# Stochastic and deterministic processes in Asymmetric Tsetlin Machine

**DOI:** 10.3389/frai.2025.1377944

**Published:** 2025-06-20

**Authors:** Negar Elmisadr, Mohamed-Bachir Belaid, Anis Yazidi

**Affiliations:** ^1^Department of Computer Science, Faculty of Technology, Art and Design, OsloMet–Oslo Metropolitan University, Oslo, Norway; ^2^NILU, Climate and Environmental Research Institute, Oslo, Norway; ^3^Department of Computer Science, Faculty of Technology, Art and Design, OsloMet–Oslo Metropolitan University, Oslo, Norway

**Keywords:** Tsetlin Machine (TM), Stochastic Point Location (SPL) algorithm, decaying normal distribution function, probabilistic and deterministic behavior, Asymmetric Tsetlin (AT) Machine, Asymmetric Probabilistic Tsetlin (APT) Machine, cumulative distribution function (CDF)

## Abstract

This paper introduces a new approach to enhance the decision-making capabilities of the Tsetlin Machine (TM) through the Stochastic Point Location (SPL) algorithm and the Asymmetric Steps technique. We incorporate stochasticity and asymmetry into the TM's process, along with a decaying normal distribution function that improves adaptability as it converges toward zero over time. We present two methods: the Asymmetric Probabilistic Tsetlin (APT) Machine, influenced by random events, and the Asymmetric Tsetlin (AT) Machine, which transitions from probabilistic to deterministic states. We evaluate these methods against traditional machine learning algorithms and classical Tsetlin (CT) machines across various benchmark datasets. Both AT and APT demonstrate competitive performance, with the AT model notably excelling, especially in complex datasets.

## 1 Introduction

Artificial Intelligence (AI) and Machine Learning (ML) techniques have transformed the field of pattern classification. Pattern classification involves the process of assigning a label or category to an input based on its features. This has numerous applications in computer vision, speech recognition, natural language processing, and more (Escobar and Morales-Menendez, 2018). Machine learning algorithms are well-suited for pattern classification tasks, as they can learn to recognize patterns in data. This is achieved by analyzing large amounts of training data and extracting features that are relevant to the classification task. Once the features have been extracted, the machine learning algorithm can use them to make predictions about new unseen data (Sarker, 2021). The ability of machine learning algorithms to learn from data and make predictions has led to numerous applications in various domains, and the continued development of machine learning algorithms is expected to advance the field of pattern classification further. Recent advancements in adversarial attacks highlight the complexity of securing deep learning systems. Hyun Kwon et al. have contributed significantly to this field, proposing innovative attack and defense methods. In Kwon (2023), Kwon introduced color-weighted distortions for image perturbations, while in Kwon and Kim (2023), a dual-mode method for generating adversarial examples was developed. These techniques were extended to specialized domains, such as helicopter recognition systems (Lee et al., 2024) and speech recognition, where multi-targeted audio adversarial attacks (Ko et al., 2023) and detection mechanisms using style transfer (Kwon et al., 2023) and classification scores (Kwon and Nam, 2023) were explored. Additionally, Kwon and Lee addressed textual adversarial examples using text modification strategies (Kwon and Lee, 2023). Together, these studies demonstrate the multifaceted nature of adversarial threats and provide insights into enhancing AI system resilience across modalities.

The introduction of learning automata has further enhanced the capabilities of AI and ML in pattern classification (Barto and Anandan, 1985). Learning automata are a type of artificial intelligence that can learn and adapt to their environment. They can make decisions based on feedback from their environment and adjust their behavior accordingly. In the context of classification tasks, learning automata can be used to optimize the parameters of a classification model based on feedback from the data, improving its performance over time Guo et al. (2017). Recognizing that decision-making involves a broad spectrum of challenges, including sequential processes and dynamic environments, the current work aims to focus on establishing fundamental mechanisms through predictive modeling tasks. Hence, the presented foundational approach is expected to allow us to validate the core principles of asymmetric transitions before extending them to more complex decision-making scenarios. The stochastic and deterministic elements we introduce naturally align with requirements for sequential decision-making, where the balance of exploration and exploitation becomes increasingly critical as environmental complexity grows.

One type of learning automata is the Tsetlin Automaton (TA), which Michael Tsetlin first introduced in the 1960s (Omslandseter et al., 2022). The Tsetlin Automata are specifically designed for pattern recognition tasks and can effectively classify input data by identifying inherent patterns within the data. These automata function by deconstructing the patterns into distinct components and then employing a set of automata to accurately recognize each of these components (Granmo et al., 2019). Tsetlin Automata are inherently interpretable, which means that it is possible to understand how the classification decision was made (Granmo, 2018; Abeyrathna et al., 2021). The Tsetlin Machine(TM) is an extension of the Tsetlin Automaton and a recent addition to the field of TA. The TM is a rule-based machine learning that extracts human-interpretable patterns from data using propositional reasoning. It is specifically designed for binary classification tasks and has exhibited promising results in handling complex pattern recognition challenges (Abeyrathna et al., 2019; Przybysz et al., 2023; Saha et al., 2023). Several studies have explored the use of Tsetlin Machine for pattern recognition tasks. TM has shown promising results in various applications, including image classification, text classification, and speech recognition. Phoulady et al. (2019), evaluated the performance of Tsetlin Machine in image classification tasks. The authors compared the results of Tsetlin automata with other classification algorithms, including artificial neural networks and support vector machines. They found that Tsetlin Machine performed competitively with the other algorithms. Bhattarai et al. (2022), explored the use of Tsetlin Machine for text classification tasks. The authors proposed a modified version of Tsetlin Machine that could handle text data directly. They evaluated the performance of their proposed algorithm on several benchmark datasets and found that it outperformed several other classification algorithms. In a different application, Bakar et al. (2022), used Tsetlin Machine for speech recognition tasks. The authors proposed a hybrid approach that combined Tsetlin automata with deep learning models. They evaluated their approach on a speech recognition dataset and found that it achieved competitive accuracy rates compared to traditional deep learning models. Additionally, several studies have explored the use of different types of reinforcement learning techniques to improve the learning process of TA. Rahimi Gorji et al. (2021) introduced a reinforcement learning approach to TA that improved its performance on several benchmark datasets. However, these approaches have limitations when dealing with complex patterns in noisy and high-dimensional data. Furthermore, the Tsetlin machine is typically trained using a different approach called the Tsetlin Automata Learning (TAL) algorithm. This algorithm possesses the capability to learn from both positive and negative feedback, rendering it highly compatible with reinforcement learning endeavors. In reinforcement learning, the agent learns to interact with the environment and take the reward over time by selecting actions based on its current state (Nowé et al., 2025). Deterministic policies include straightforward decision-making and ease of interpretation, making them a preferred choice in certain scenarios. while probabilistic policies can capture uncertainty and provide more flexibility in decision-making (Lecerf, 2022; Cox, 2012).

Stochastic Point Location (SPL) is a technique used in reinforcement learning to explore action spaces efficiently. SPL is a computational algorithm that helps an agent to locate a point on a line in an environment. It involves randomly selecting a point within the action space and taking an action based on that point. Repetition of this process multiple times enables the agent to explore different regions of the action space while also exploiting the known regions, striking a balance between exploration and exploitation (Abolpour Mofrad et al., 2019; Gullapalli, 1990). It is designed to help the agent efficiently search for the optimal action by reducing the number of computational resources required for exploration (Yazidi et al., 2012). By improving exploration efficiency, SPL can help reinforcement learning agents quickly find optimal solutions to complex problems. In general, the SPL algorithm is a probabilistic search algorithm that can be widely used in machine learning tasks. SPL can be used to generate features or identify important regions of an image or object, which can then be used for downstream tasks such as classification or segmentation (Zhang et al., 2022; Yazidi et al., 2017; Hosseinijou and Bashiri, 2012). In more detail, Haran and Halperin (2009) presented an efficient algorithm for locating a query point in a planar subdivision, such as a Voronoi diagram or Delaunay triangulation. The algorithm is based on a Stochastic Point Location (SPL) technique, which involves randomly walking from a starting point to a point in the subdivision that is close enough to the query point. Granmo and Oommen proposed an approach based on SPL to address resource allocation problems in noisy environments in their work (Granmo and Oommen, 2010). This method calculates the probability of polling a resource from two available options at each time step. In the field of cybersecurity, SPL is employed to represent deviations from expected behaviors, facilitating the efficient detection of network anomalies, as described in Karthik (2017). SPL has been significantly modified in Guo et al. (2016) to align with the Random Walk-based Algorithm. These modifications enhance the algorithm's performance and broaden its applicability, making it more effective in various scenarios. The hierarchical SPL scheme has been broadened and made more general in Zhang et al. (2006). This evolution of the algorithm expands its capabilities and makes it more adaptable to a wider range of tasks and environments, further enhancing its utility and relevance. Grady (2006) present a fast algorithm for segmenting images using random walks. The algorithm uses a graph representation of the image, where each pixel is a node, and the edges connect neighboring pixels. A random walk process is used to propagate labels from a seed point to the rest of the image, and the resulting label probabilities are used to segment the image. Tong and Faloutsos (2006) present a technique for identifying “center-piece” subgraphs in large networks. These subgraphs represent the most influential or central nodes in the network and are useful for tasks such as community detection and recommendation. The technique is based on a random walk process, where a random walker starts from a random node and moves around the network with the goal of visiting nodes that have high centrality.

Yazidi and Oommen (2017), proposed a new variant of SPL, to find the root of a stochastic equation, where the equation may have one or more unknowns and the solution is found through a process of iterative search and pruning using an adaptive d-ary search approach. In their approach, they consider a non-constant probability and non-symmetry responses of the “environment”, which makes a more accurate and efficient approach to stochastic root-finding problems than in previous cases where probability was assumed to be constant and symmetric.

Motivation and contribution: Our investigation into enhancing the Tsetlin Machine (TM) is driven by several fundamental limitations in existing approaches. The traditional TM employs symmetric state transitions, which can lead to inefficient exploration of the state space, particularly in complex pattern recognition tasks. This symmetry may restrict the model's ability to adapt to varying levels of feedback importance. Moreover, the deterministic nature of conventional TM state transitions can cause the model to become trapped in suboptimal states, especially when faced with noisy or inconsistent feedback patterns. Current TM implementations also lack mechanisms for dynamically balancing exploration and exploitation, which are essential for optimal learning in complex environments.

To address these limitations, we introduce two novel approaches: the Asymmetric Tsetlin (AT) Machine and the Asymmetric Probabilistic Tsetlin (APT) Machine. Our methods represent a significant advancement in Tsetlin Machine (TM) theory through several key scientific contributions. First, we introduce an enhanced learning dynamic that incorporates asymmetric transition mechanisms. This allows for more nuanced exploration of the state space, utilizing variable step sizes in different directions, which facilitates faster adaptation to important patterns. Second, we present theoretical advancements through a mathematically rigorous framework for asymmetric state transitions. This framework combines probabilistic and deterministic processes using a decaying normal distribution with proven convergence properties. Finally, we demonstrate practical improvements, including reduced sensitivity to initial state configurations, more efficient handling of imbalanced feedback, and enhanced adaptation to complex pattern recognition tasks. Classical Tsetlin Machines (TMs) are interpretable and symbolic, but they rely on symmetric, deterministic transitions, which limit their adaptability in noisy, complex, or dynamically changing environments. The introduction of Asymmetric Tsetlin (AT) and Asymmetric Probabilistic Tsetlin (APT) Machines aims to overcome this rigidity by incorporating directional bias and controlled randomness. These enhancements create a more sophisticated balance between exploration and exploitation, improving the robustness of learning and the efficiency of convergence. Importantly, AT and APT maintain the interpretability advantages of classical TMs, representing a significant scientific advancement within symbolic and interpretable learning paradigms. Our empirical evaluation, using established benchmark datasets, supports the validity of this theoretical innovation under controlled and reproducible conditions.

The scientific significance of our work lies in the novel mathematical framework that bridges the gap between purely deterministic and probabilistic approaches. This hybrid nature allows our model to maintain the interpretability advantages of classical TMs while incorporating the flexibility and robustness of probabilistic methods. Through extensive evaluation of multiple benchmark datasets, we demonstrate that these theoretical advantages translate into practical performance improvements, particularly in scenarios involving complex pattern recognition tasks or noisy data environments.

Our main contributions can be summarized as follows: (1) we developed a novel asymmetric transition mechanism that improves state space exploration, (2) we integrated stochastic and deterministic processes through a sound mathematical framework, (3) we introduced a dynamic balance between exploration and exploitation by using controlled randomness, and (4) we empirically validated performance improvements across various benchmark datasets. The asymmetric transition mechanism presented here has been validated for predictive tasks and has broader implications for sequential decision-making. In addition, the combination of stochastic and deterministic processes interestingly addresses temporal dependencies and varying environmental states, which can suggest promising applications in reinforcement learning and open up new research opportunities.

**Figure 1:**
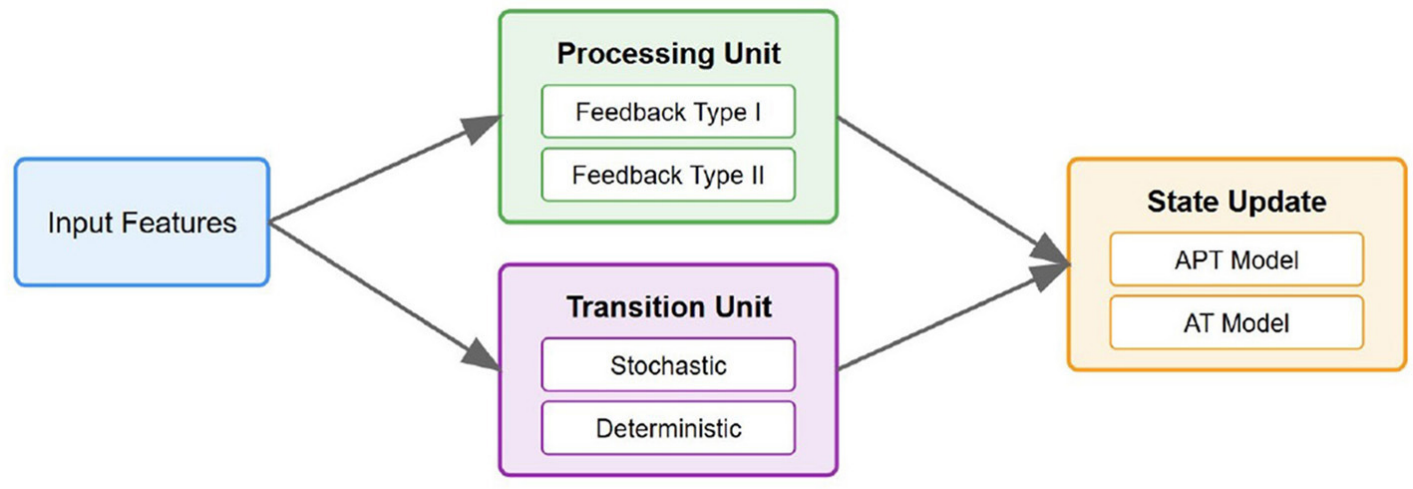
The architecture of the proposed AT/APT framework.

To provide further clarity, Figure 1 illustrates the architecture of the proposed AT/APT framework. This framework processes input features through a processing unit that manages both Type I and Type II feedback mechanisms. The transition unit incorporates both stochastic and deterministic components, allowing for adaptive state transitions. Meanwhile, the state update component implements both APT and AT models; APT maintains probabilistic transitions, whereas AT integrates both stochastic and deterministic elements.

The paper is organized as follows: Section 2 offers a detailed explanation of the Materials and Methods, covering the fundamental concepts of Tsetlin Automata (TA) and Tsetlin Machine (TM), including their architecture and learning processes. It also outlines the approach taken in this study, which involves integrating an asymmetric and stochastic method with the SPL algorithm into Tsetlin Automata and examines strategies to determine transition probabilities between states. Section 3 highlights the key contributions where our new paradigm is introduced. Section 4 presents the evaluation results obtained from applying the proposed approach to various datasets. Section 5 discusses prospective future work that can build upon this framework. Finally, Section 6 concludes the paper and emphasizes the main achievements of the proposed approach.

## 2 Materials and methods

### 2.1 Background

### 2.2 Stochastic point location

The Stochastic Point Location (SPL) problem, sometimes called Stochastic Search on the Line, involves a learning mechanism (LM) to find the optimal value of a parameter λ. We assume an unknown optimal choice λ^*^ and aim to study the learning process. The LM tries to determine λ with feedback from an intelligent environment (E), indicating if λ is too small or too large. The environment's responses are stochastic. It might give incorrect feedback, suggesting changes opposite to what's needed. The probability of getting correct feedback must be larger than 0.5 (i.e., *p*>0.5) for λ to converge to λ^*^. This probability represents the environment's “effectiveness.” When λ is less than λ^*^, the environment correctly suggests raising λ with probability p, but might wrongly suggest lowering it with probability (1 - p). Similarly, when λ is greater than λ^*^, the environment may suggest lowering λ with probability p and raising it with probability (1 - p). Further details of the SPL algorithm can be found in Oommen (1997).

### 2.3 Tsetlin automata

Tsetlin automata (TA) uses a memory array to store a set of rules that determine the output based on the input signals. A simple two-action Tsetlin Automaton is defined by the quintuple:

ϕ, α, β, *F*(·, ·), *G*(·).

ϕ: set of internal states (ϕ = ϕ_1_, …, ϕ_2*N*_.

α: set of automaton actions (α = α_1_, α_2_).β: a set of the feedback given to the automaton in terms of reward and penalty.

*F*(ϕ_*t*_, β_*t*_): transition function, determining the internal state of the automaton.

*G*(ϕ_*t*_):output function, determining the action (α_*t*_) performed by the automaton given the current state (ϕ_*t*_).

TA performs actions in a stochastic environment. By interacting with the environment, it aims to learn the optimal action, i.e., the one that has the highest probability of eliciting a reward.

### 2.4 Tsetlin Machine

**Figure 2:**
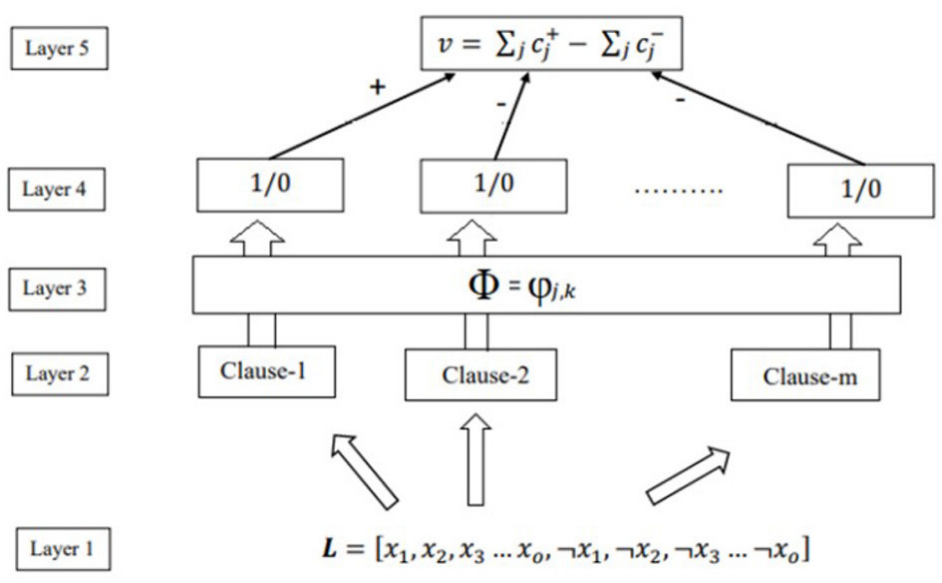
Structure of TM.

The Tsetlin Machine(TM) consists of multiple Tsetlin automata that are organized in layers. Figure 2 shows the architecture of TM, conceptually decomposed into five layers to recognize subpatterns in the data and categorize them into classes (Abeyrathna et al., 2022). The function of each of these layers is described in the following.

**Input layer:** The first layer of the Tsetlin Machine consists of a set of binary features or input variables, which are represented as literals (positive statements) and negated literals (negative statements). The input variables can be represented as follows:

Let *X* = *x*_1_, *x*_2_, *x*_3_, …, *x*_*n*_ be a set of binary input variables *n*, where *x*_*i*_ can take the values 0 or 1. Each input variable *x*_*i*_ can be represented as a literal or a negated literal as follows: *x*_*i*_ for a literal and ¬*x*_*i*_ for its negation.

Collectively, the elements in the augmented feature vector are as follows:


$$L=\left[x_{1},x_{2},\ldots ,x_{n},\neg x_{1},\neg x_{n},\ldots ,\neg x_{n}\right]=\left[l_{1},l_{2},\ldots ,l_{2o}\right]$$

These literals and negated literals can be combined using logical operators such as AND, OR, and NOT to form logical expressions. The first layer uses these logical expressions as clauses to represent patterns in the data.

**Clauses layer:** There are m conjunctive clauses that capture the subpatterns associated with classes 1 and 0. The value *m* is set by the user. Accordingly, given that more complex problems might require larger *m*. All the clauses receive the same augmented feature vector L, assembled at the input layer. However, to perform the conjunction, only a fraction of the literals are selected, with the conjunction performed as follows:


(1) $$\begin{array}{l} c_{j}=\underset{k\in Ij}{\wedge }l_{k} \end{array}$$

Notice how the composition of a clause varies from another clause depending on the indexes of the included literals in the set *I*_*j*_ ⊆ 1, …, 2*o*.

**State layer:** The State Layer in the Tsetlin machine architecture stores the states of the clauses in the memory matrix and updates those states while processing an input pattern. The state update rule depends on the input pattern and the bias parameters associated with each clause, which can be learned during training.

**Clause output layer:** Once the TA decisions are available, the output of the clause can be easily calculated. Since clauses are conjunctive, a single literal of value 0 is enough to convert the output of the clause to 0 if its corresponding TA has decided to include it in the clause. For ease of understanding, we introduce the set $I_{x}^{1}$, which contains the indexes of the literals of value 1. Then, the output of clause j can be expressed as:


(2) $$c_{j}=\{\begin{array}{ll} 1, & \text{if }I_{j}\subseteq I_{x}^{1}\\ 0, & \text{otherwise} \end{array}$$

The clause outputs, computed as above, are now stored in the vector **C**, i.e., $\text{C}=\left(c_{j}\right)\in {0,1}^{m}$.

**Classification layer:** The TM structure is shown in Figure 2—classifies data into two classes. Therefore, the subpatterns associated with each class have to be learned separately. For this purpose, the clauses are divided into two groups, where one group learns the subpattern of class 1 while the other learns the subpatterns of class 0. For simplicity, clauses with odd indexes are assigned with positive polarity ($c_{j}^{+}$), and they are supposed to capture subpatterns of output *y* = 1. Clauses with even index, on the other hand, are assigned with negative polarity ($c_{j}^{-}$), and they are supposed to capture subpatterns of output *y* = 0—the clauses which recognize subpattern output 1. We need to sum each class's clause outputs and assign the sample to the class with the highest sum. A higher sum means that more sub-patterns have been identified from the designated class, and the input sample has a higher chance of being of that class. Hence, with v being the difference in clause output.


(3) $$\begin{array}{l} v=\underset{j}{\sum }c_{j}^{+}-\underset{j}{\sum }c_{j}^{-} \end{array}$$

The output of the TM is decided as follows:


(4) $$\hat{y}=\{\begin{array}{ll} 1, & v\geq 0\\ 0, & v<0 \end{array}$$

#### 2.4.1 **Learning procedure**

In classical Tsetlin machine, learning which literals to include is based on two types of reinforcement: Type I feedback and Type II feedback. Type I feedback aims to reduce false negatives by reinforcing correct actions, while Type II feedback increases the discrimination power of the patterns.

##### 2.4.1.1 **Type I feedback: reduce false negatives**

Type I feedback is formulated to enhance the true positive outputs of clauses while mitigating false negative outputs. In order to bolster a clause's true positive output (where the clause output should be 1), Type I Feedback reinforces the “Include” actions of Tsetlin Automata (TAs) corresponding to literal values of 1. Concurrently, within the same clause, Type I Feedback amplifies the “Exclude” actions of TAs linked to literal values of 0. To address instances of incorrect negative clause outputs (where the clause output should be 0), a gradual erasure of the currently identified pattern is initiated. This is executed by intensifying the “Exclude” actions of TAs, irrespective of their corresponding literal values. As a result, clauses with positive polarity necessitate Type I feedback when *y* = 1, while clauses with negative polarity demand Type I feedback when *y* = 0. Further detail can be found in in Abeyrathna et al. (2022).

##### 2.4.1.2 **Type II feedback**

Type II feedback aims to reduce false positive clause outputs. It focuses on turning a clause output from 1 to 0 when it should be 0. This feedback type includes a literal of value 0 in the clause to achieve this. Clauses with positive polarity need Type II feedback when the desired output (y) is 0, and clauses with negative polarity need it when y is 1, as they do not want to vote for the opposite class.

the procedure outlined for Type II feedback can be summarized in Table 1.

**Table 1:** Type II feedback in Tsetlin Machine.

	**Clause value**	**1**	**1**	**0**	**0**
	**Literal value**	**1**	**0**	**1**	**0**
Include literal:	*P* (reward)	0	NA	0	0
	*P* (inaction)	1	NA	1	1
	*P* (penalty)	0	NA	0	0
Exclude literal:	*P* (reward)	0	0	0	0
	*P* (inaction)	1	0	1	1
	*P* (penalty)	0	1	0	0

## 3 Contribution

### 3.1 Asymmetric stochastic point location

The Asymmetric-SPL method presents a variation of the Stochastic Point Location (SPL) approach, introducing an asymmetric transition rule that allows for distinct step sizes when moving in the right and left directions. This asymmetry is quantified by two integers, denoted as “a” and “b”, which signify the number of steps taken when moving right and left, respectively, thereby capturing the directional bias inherent in the environment. Let us denote the probability of moving to the right is function of λ i.e., *p*[λ(*n*)], and correspondingly, the probability of moving to the left as 1 - *p*[λ(*n*)]. The update rule governing these transitions is formulated as follows:

For a suggested movement of “a” steps to the right by the environment E with a probability of *p*[λ(*n*)], the parameter λ at time step n + 1 is updated as:


(5) $$\begin{array}{l} \lambda \left(n+1\right)\leftarrow \lambda \left(n\right)+a \end{array}$$

Conversely, when E suggests 'b' steps to the left with a probability of 1 - p(λ(*n*)), the update for λ becomes:


(6) $$\begin{array}{l} \lambda \left(n+1\right)\leftarrow \lambda \left(n\right)-b \end{array}$$

When a transition occurs with a probability of *p* to move toward the right, the anticipated number of rightward steps over numerous transitions is calculated as *a*×*p*. This value is derived from the average quantity of steps moved rightward per transition. By multiplying this value with the total count of transitions, the projected number of rightward steps can be approximated. Similarly, when a transition takes place with a probability of 1−*p* to move toward the left, the anticipated number of leftward steps following a substantial number of transitions becomes *b*×(1−*p*). This corresponds to the mean number of steps moved leftward per transition. By multiplying this value with the total transition count, the projected number of leftward steps can be estimated.

Given the distinct probabilities of moving right and left at the optimal point within the Asymmetric-SPL algorithm, it is essential to ensure equilibrium by making the total number of rightward steps equal to the total number of leftward steps. This is necessary due to the prevailing directional preference or bias in the environment, causing the system to exhibit a greater inclination toward one direction over the other.

To satisfy this equilibrium condition, we can establish the following relationship:


(7) $$\begin{array}{l} a\times p=b\times \left(1-p\right). \end{array}$$

This equation signifies that the projected number of steps moved toward the right should be equivalent to the projected number of steps moved toward the left, thus ensuring a harmoniously balanced system.

### 3.2 Asymmetric Tsetlin automata

**Figure 3:**
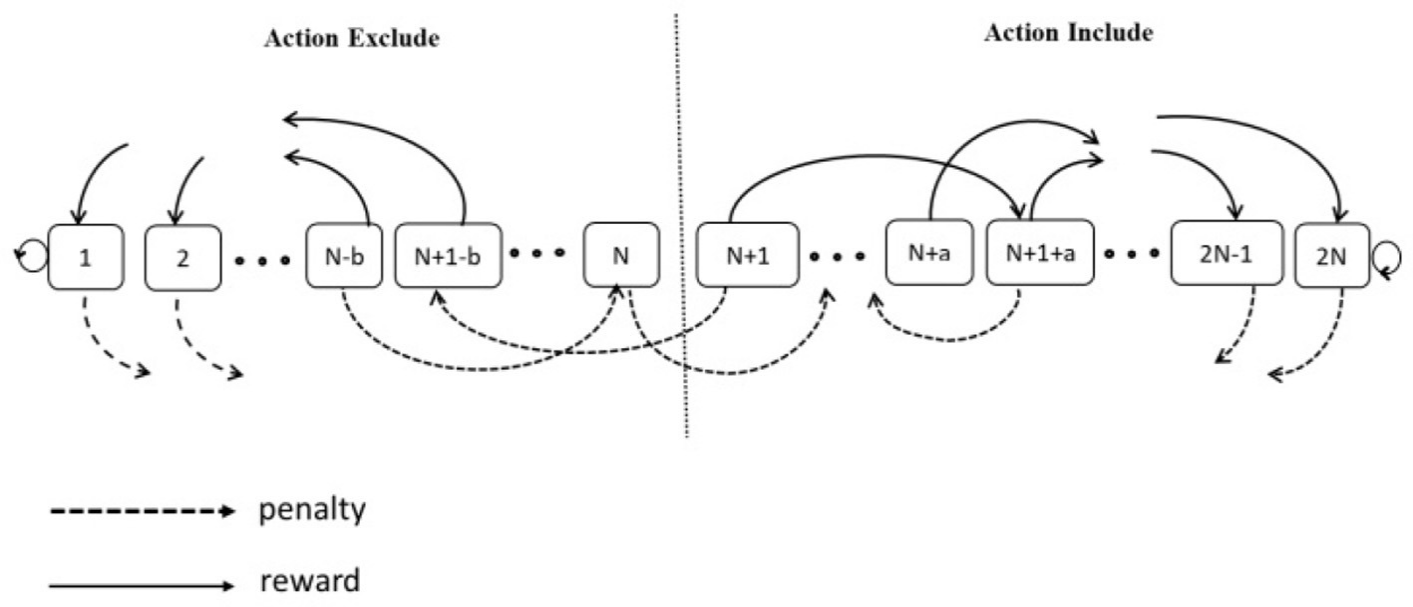
A two-action Tsetlin automata with the asymmetric steps of transition.

The schematic of the Asymmetric transition between the states of Tsetlin automata is drawn in Figure 3.

The illustration depicts a two-action Tsetlin Automaton (TA) characterized by 2*N* memory states, where *N* is a variable spanning the range [1, ∞). Within the state range from 1 to *N*, situated on the left-hand side of the state space, the TA selects Action 1 (Exclude). Conversely, when the system resides in the state range from *N* + 1 to 2*N”*, positioned on the right side of the state space, the TA's choice is Action 2 (Include). In each interaction with the environment, the TA executes one of the available actions.

By using the asymmetric SPL method to change the states of the Tsetlin Automata, the estimated form provided by the algorithm would be used to determine the action taken by the Tsetlin Automata. Once the action is taken, the Tsetlin Automata receives feedback as a reward or penalty, and its state is updated accordingly. If the TA gets a penalty, it will move “b” steps toward the opposite side of the current action. On the contrary, if the TA receives a reward as a response, it will switch to a “deeper” state by moving “a” steps to the left or right end of the state chain, depending on whether the current action is Action 1 or Action 2.

### 3.3 Asymmetric Tsetlin Machine

The Asymmetric Tsetlin Machine is a variant of the Tsetlin Machine (TM) that utilizes the Asymmetric Tsetlin Automaton as its fundamental building block. Featuring an expanded learning procedure with modified Type I feedback and standard Type II feedback. The modifications to Type I feedback involve transition probability adjustments designed to enhance performance.

### 3.4 Learning procedure

#### 3.4.1 Enhanced learning procedure

The Asymmetric Tsetlin Machine employs an enhanced learning mechanism that utilizes modified transitions in Type I feedback. These modifications are essential to enhance performance by minimizing false negatives. Type II feedback operates with a distinct objective; the mere incorporation of a literal with a value of 0 within the clause proves adequate for achieving the desired reduction in false-positive clause outputs. Therefore, Type II feedback does not necessitate these particular modifications.

##### 3.4.1.1 Modified type I feedback

The classical Tsetlin machine incorporates an inaction probability, which indicates the likelihood of Tsetlin Automata remaining in their present states. To achieve an asymmetric transition, crucial for enhanced performance, the inaction probability is replaced by reward or penalty probabilities, depending on which one is most likely. Eliminating the inaction probability introduces an asymmetry in the transition probabilities. Consequently, the probability of transitioning from one state to another becomes imbalanced. This imbalance stems from the distinction in transitioning likelihoods based on whether an action encompasses or excludes a specific clause. The result is a dynamic shift in the machine's behavior, impacting the system's responsiveness and adaptability during its operation. Table 2 displays the modified feedback type I table with the necessary adjustments for achieving asymmetric transitions.

**Table 2:** Modified type I feedback in Tsetlin Machine.

	**Clause value**	**1**	**1**	**0**	**0**
	**Literal value**	**1**	**0**	**1**	**0**
Include literal:	*P* (reward)	(*s*−1)/*s*	NA	1/*s*	1/*s*
	*P* (penalty)	1/*s*	NA	(*s*−1)/*s*	(*s*−1)/*s*
Exclude literal:	*P* (reward)	1/*s*	(*s*−1)/*s*	(*s*−1)/*s*	(*s*−1)/*s*
	*P* (penalty)	(*s*−1)/*s*	1/*s*	1/*s*	1/*s*

The value *s* is a user-defined parameter.

##### 3.4.1.2 **Transition probability**

The behavior of the Tsetlin Automata in response to input features is significantly influenced by the transition probabilities between its states, which play a critical role. These probabilities hinge on various factors, including the current state of the Tsetlin Automata, the literal value, and the current clause value. However, there is a potential vulnerability in Tsetlin machines where they might become trapped in suboptimal states if their exploration of the state space is insufficient.

To mitigate this issue, the introduction of randomness into state transitions emerges as a viable solution. This adjustment allows the Tsetlin machine to traverse through diverse states, thereby preventing it from becoming trapped in unfavorable states due to inadequate exploration. The incorporation of randomness is achieved through the utilization of a standard normal (standard Gaussian) random variable denoted as ϵ. This variable possesses an average of zero and its standard deviation diminishes as epochs progress, a trend determined by the exponential function.


(8) $$\begin{array}{l} \epsilon \sim N\left(0,\sigma \left(e_{i}\right)\right),\;\text{where }\sigma \left(e_{i}\right)=\sigma \left(0\right)\exp\left(-d.e_{i}\right) \end{array}$$

where σ(0) represents the initial standard deviation, *d* stands for the rate of decay, and *e*_*i*_ corresponds to the *i*^*th*^ epoch. This formulation signifies that the standard deviation of randomness decreases over epochs, resulting in a reduction of randomness in state transitions over time. Consequently, the Tsetlin machine gradually shifts its focus from exploration toward exploiting the optimal state.

Through the incorporation of this decaying standard deviation-based randomness, the Tsetlin machine effectively balances its exploration and exploitation tendencies. This equilibrium is achieved by introducing variability that is inversely proportional to the progression of epochs, thus adapting the machine's behavior over time.

**Learning mechanism** in the Asymmetric Tsetlin Machine is governed by probabilistic and deterministic transition mechanisms. These mechanisms ensure the model's adaptability and robustness in a biased environment. We'll explore two methods. The first one uses random transitions between states, and we'll call it Asymmetric Probabilistic Tsetlin (APT) automata. The second method, on the other hand, makes transitions more certain and definite, and we'll name it Asymmetric Tsetlin (AT) automata, which blends elements of both probabilistic and deterministic processes within the Tsetlin Automata framework.

(a) Probabilistic transitions:

In the stochastic or probabilistic method, we define the updated probabilities as follows:


(9) $$\begin{array}{l} P_{new}(reward{)}_{APT}=p(reward)+N(0,exp(-d.e_{i})\\ P_{new}(penalty{)}_{APT}=p(penalty)+N(0,exp(-d.e_{i}) \end{array}$$

As observed, it is based on the classical Tsetlin automata used in classical Tsetlin Machine, a transition between states is determined based on a comparison between the probability of a reward and the probability of a penalty with a randomly generated number, This introduces a probabilistic element into the decision-making process, where random events influence the outcome of a decision.

(b)Deterministic transitions:

In the deterministic method, we compare the likelihood probabilities directly. Conducting a meticulous comparison of likelihood probabilities enables us to derive quantitative insights into the relative occurrences of specific events. This analytical approach facilitates informed decision-making based on accurate probability values. To assess whether the likelihood of receiving a reward surpasses that of incurring a penalty (i.e., *P*(*P*(*reward*)>*P*(*penalty*)), or conversely, whether the likelihood of a penalty outweighing the reward exists (i.e., *P*(*P*(*penalty*)>*P*(*reward*)), we employ a well-defined methodology. This approach, which considers the actual probability values, becomes particularly relevant when our objective is to comprehensively understand the interplay between these events. The assessment is accomplished through a specific procedure involving statistical measures, notably the utilization of the cumulative distribution function (CDF) for the normal distribution.

As we proceed with our procedure, we can consider updated probabilities for both reward and penalty as follows:


(10) $$\begin{array}{l} P_{new}(reward{)}_{AT}=P(p(reward)+N(0,exp(-d.e_{i}))>p(penalty)\\ \;+N(0,exp(-d.e_{i}))\\ P_{new}(penalty{)}_{AT}=P(p(penalty)+N(0,exp(-d.e_{i}))>p(penalty)\\ \;+N(0,exp(-d.e_{i})) \end{array}$$

Upon observation from Table 2, it becomes apparent that we have precisely two distinct values for the probabilities, namely $\frac{\left(s-1\right)}{s}$ and $\frac{1}{s}$. These probabilities can be represented as numerical values by assigning a specific value to “s” Let's define the variables as follows:


$$\begin{array}{l} X=\frac{\left(s-1\right)}{s}+N\left(0,exp\left(-d.e_{i}\right)\right)\\ Y=\frac{1}{s}+N\left(0,exp\left(-d.e_{i}\right)\right) \end{array}$$

To calculate the *p*(*X* > *Y*), we can leverage the property that the difference between two independent normal random variables follows a normal distribution. This allows us to easily calculate its mean and variance based on the means and variances of the original random variables. The mean and variance of X are:


$$\begin{array}{l} mean\left(X\right)=\left(s-1\right)/s+mean\left(N\left(0,exp\left(-d*e_{i}\right)\right)\right)=\left(s-1\right)\\ var\left(X\right)=var\left(N\left(0,exp\left(-d.ei\right)\right)\right)=exp\left(-2d.e_{i}\right) \end{array}$$

Similarly, the mean and variance of Y are:


$$\begin{array}{l} mean\left(Y\right)=1/s+mean\left(N\left(0,exp\left(-d*e_{i}\right)\right)\right)=1/s\\ var\left(Y\right)=var\left(N\left(0,exp\left(-d.e_{i}\right)\right)\right)=exp\left(-2d.e_{i}\right) \end{array}$$

Now, we can find the mean and variance of the difference *Z* = *X*−*Y* as follows:


$$\begin{array}{ll} mean\left(Z\right)=mean\left(X\right)-mean\left(Y\right)=\left(s-1\right)/s-1/s\\ =\left(s-2\right)/s\\ var\left(Z\right)=var\left(X\right)+var\left(Y\right)=2exp\left(-2d.e_{i}\right)\\ \; & \; \end{array}$$

Therefore, Z is a normal random variable with mean(z) and var(z).Now, we can calculate the probability of the inequality as follows:


$$\begin{array}{ll} P\left(X-Y>0\right)=P\left(Z>0\right)\\ \; & \; \end{array}$$

Using the mean and variance of Z, we can standardize it by subtracting its mean and dividing by its standard deviation:


$$\begin{array}{l} Z_{standardized}=\left(Z-mean\left(Z\right)\right)/\sqrt{var\left(Z\right)} \end{array}$$

Substituting the values of mean(Z) and var(Z), we get:


$$Z_{standardized}=(X-Y-(s-2)/s/\sqrt{2exp(-2d.e_{i})}$$

Now, we can rewrite the inequality as:


$$\begin{array}{l} Z_{standardized}>\left(s-2\right)/s/\sqrt{2exp\left(-2d.e_{i}\right)} \end{array}$$

Finally, we can use the cumulative distribution function (CDF) of the normal distribution to calculate the probability:


$$\begin{array}{l} P\left(X-Y>0\right)=P\left(Z>0\right)\\ =P\left(Z_{\text{standardized}}>\frac{\left(s-2\right)/s}{\sqrt{2\exp\left(-2d\cdot e_{i}\right)}}\right)\\ =1-\Phi \left(-\frac{\left(s-2\right)/s}{\sqrt{2\exp\left(-2d\cdot e_{i}\right)}}\right) \end{array}$$

Where ϕ is the CDF of the standard normal distribution. Hence, we can state the following:


(11) $$\begin{array}{l} P\left(\frac{s-1}{s}+N\left(0,\exp\left(-d\cdot e_{i}\right)\right)>\frac{1}{s}+N\left(0,\exp\left(-d\cdot e_{i}\right)\right)\right)\\ =1-\Phi \left(-\frac{\left(s-2\right)/s}{\sqrt{2\exp\left(-2d\cdot e_{i}\right)}}\right)\\ =\alpha_{i} \end{array}$$

The parameter α_*i*_ implies a likelihood of receiving a reward in Asymmetric Tsetlin Machines (AT) at epoch i. Mathematically, we observe that the decaying normal distribution function approaches zero as epochs or time tends to infinity. This convergence toward zero signifies that, in the long run, when setting the “s” parameter precisely, the model transitions from a probabilistic to a deterministic state, causing α in Equation (11) to approach 1. This dynamic modeling framework harmonizes probabilistic adaptability with deterministic stability.

With reference to Table 2 and in light of Equation 7, the state update protocol for Tsetlin Automata can be deduced by taking into account the feedback sourced from the environment and the consequent action associated with the respective clause. These protocols are subsequently consolidated in Table 3. To clarify the individual contributions of each component, the theoretical isolation is presented in the following based on the achieved logic and observed results. The SPL algorithm enhances exploration through stochastic transitions, the asymmetric step logic allows directional convergence, and the decaying normal distribution strategically reduces variance during learning. To avoid the need for new simulations and to gather extensive data from a formal ablation study, these design components are considered to be tested incrementally across various datasets. Their combined effect is evident in the significant improvements in accuracy that will be reported in the following.

**Table 3:** State updating rules in AT (Modified Type I Feedback).

	Clause value	1	1	0	0
	Literal value	1	0	1	0
Include Literal:	P(reward)	α_*i*_ → +*a*	NA	α_*i*_ → −*b*	α_*i*_ → −*b*
	P(penalty)	(1−α_*i*_) → −*b*	NA	(1−α_*i*_) → +*a*	(1−α_*i*_) → +*a*
Exclude Literal:	P(reward)	α_*i*_ → +*b*	α_*i*_ → −*a*	α_*i*_ → −*a*	α_*i*_ → −*a*
	P(penalty)	(1−α_*i*_) → −*a*	(1−α_*i*_) → +*b*	(1−α_*i*_) → +*b*	(1−α_*i*_) → +*b*

Intuitively, our asymmetric transitions systematically guide Tsetlin Automata toward optimal decision states, aligning well with our empirical evidence presented later. These asymmetric mechanisms, visually summarized in Table 3, clearly depict the interplay between deterministic and probabilistic behaviors, thus enhancing both interpretability and performance of the proposed models. In the Tsetlin Machine paradigm, the parameter “s”, holds paramount significance, as they intricately shape the system's behavior. Parameter “s”, often referred to as feedback strength, finely control the feedback magnitude directed at each Tsetlin Automaton during the learning phase. This adjustment process reinforces favorable decisions while discouraging the repetition of incorrect ones. Crucially, the value of “s” significantly impacts the magnitude of these adjustments. Opting for higher “s” values fosters accelerated learning but may introduce instability due to oscillatory behavior. Conversely, lower “s” values encourage a more gradual and stable learning process, albeit at a slower pace.

In the Asymmetric Tsetlin (AT) Machine, the interplay between “s”, “a”, and “b” becomes evident. The parameters “a” and “b” not only influence the rate of transition among Tsetlin Automaton states but also dictate the feedback strength. Their roles are intertwined, amplifying their collective importance. Recognizing the profound impact of these parameters on system performance, it is crucial to thoughtfully tailor “a” and “b” according to the specific problem and dataset at hand, aiming to achieve the optimal balance between exploration and exploitation.

To mitigate the challenge of parameter tuning and streamline the optimization process, we can link the number of transition steps to the variable “s”. By setting the variable “a” to the largest integer less than or equal to *s*−1, denoted as ⌊*s*−1⌋, its value can be determined.

Consequently, the value of the variable “b” can be computed using equations 7. By doing so, this method effectively simplifies both the training and optimization phases within the system. In fact, the theoretical advantages of the proposed asymmetric and probabilistic mechanisms stem from their ability to adaptively balance exploration and exploitation. Asymmetric transitions facilitate directional learning, which minimizes unnecessary back-and-forth state changes. Meanwhile, the stochastic elements of the SPL (Statistical Probabilistic Learning) enhance the model's responsiveness in uncertain or noisy conditions. These mechanisms not only improve accuracy but also provide a more robust decision-making pathway–something that conventional symmetric Tsetlin logic typically lacks.

## 4 Results

In this section, the effectiveness of our approach is evaluated by comparing it against the Classical Tsetlin machine(CT), where the Type I feedback table remains unaltered and employs symmetric state transitions with a step size of 1. This comparative analysis utilizes three widely recognized benchmark datasets: the Iris dataset, the Mushroom dataset, and the MNIST dataset. It should be noted that several factors can affect the performance of the Classical Tsetlin Machine. To ensure a fair and meaningful comparison, we kept APT, AT, and CT configured with the same settings for common parameters during the experiments. Our study focuses on the effect of transition steps in the asymmetric Tsetlin machine under our specified settings. As mentioned earlier, the parameter “s” plays a crucial role throughout both the stochastic and deterministic phases; higher values of “s” increase exploration by amplifying the stochastic elements. As the training progresses, the effect of “s” stabilizes, fine-tuning the learned states. The parameter “s” was determined through trial and error to balance exploration and exploitation effectively.

Moreover; we extend our evaluation against several other typical machine learning algorithms, including support vector machine (SVM), decision tree (DT), random forest (RF), and K-Nearest Neighbors (K-NN). The reported results in this section are obtained from 100 independent experiment trials. Each trial set involved random partitioning of the data into training (80%) and testing (20%) sets. For the normal distribution, the initial standard deviation was set to 1, while the decaying rate was set to 0.01. The source code for implementing the proposed algorithm is accessible at: https://https//github.com/elmisadr/AT

### 4.1 Iris dataset

Iris Dataset^1^ is a widely used benchmark dataset in the field of machine learning, and it contains measurements of iris flowers from three different species: setosa, versicolor, and virginica. Specifically, the dataset includes the sepal length, sepal width, petal length, and petal width of 150 iris flowers, with 50 flowers from each species. To binarize the features, we can use a threshold value to convert the continuous numerical features into binary feature labels. Upon binarizing the features and encoding the class labels, a binary dataset is generated, ready to be employed as input for the Tsetlin Machine.

**Figure 4:**
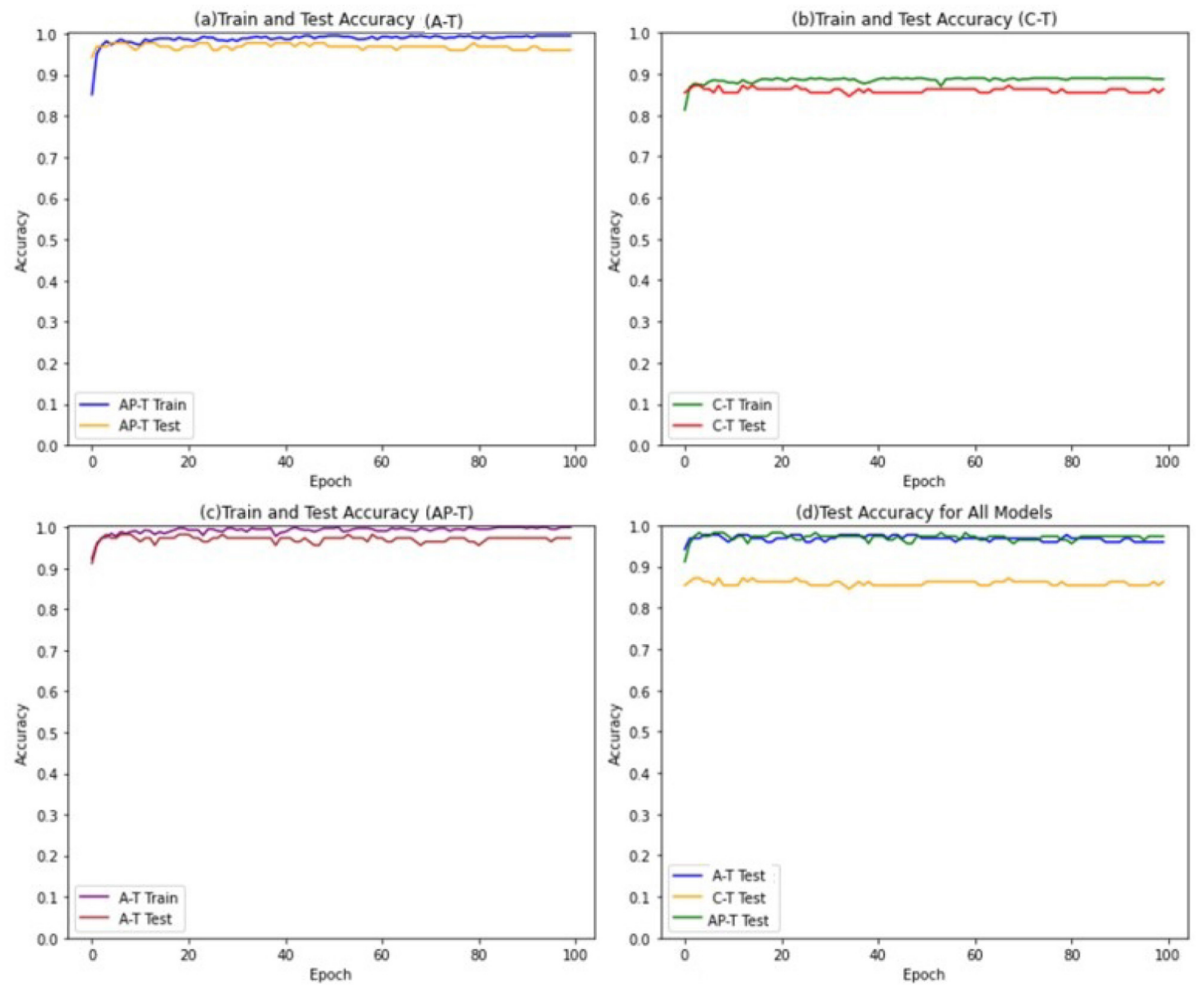
Train and test accuracies for iris dataset in AT, CT, and APT.

To achieve this, a threshold value of 0.5 is selected to convert the continuous numerical features into binary features, The Tsetlin Machine models are trained for 100 epochs, with 800 clauses and 4 features in the input data. A threshold value of 1 is used to determine the final output of the Tsetlin machine, and the s parameter is set to 4. Furthermore, the individual Tsetlin Automata each have 100 states. Figure 4 presents the performance of Asymmetric Tsetlin (AT), Asymmetric probabilistic Tsetlin (APT), and Classical Tsetlin (CT), on the iris dataset, for both the training and test data sets.

The figures show that the Asymmetric Tsetlin (AT) and Asymmetric Probabilistic Tsetlin (APT) outperform the classical Tsetlin (CT) in terms of accuracy. This is because the asymmetric transition allows for more effective exploitation of the information contained in the data. However, it is interesting to note that the Asymmetric Probabilistic Tsetlin (APT) is slightly more accurate compared to AT. This phenomenon may be attributed to the nature of the iris dataset used in the experiment, which is not particularly complex. Its simplicity and low dimensionality may not fully exploit the benefits of the more deterministic transitions introduced by the AT design. The probabilistic nature of APT, on the other hand, may offer a nuanced adaptability that proves advantageous in capturing patterns, resulting in a slight accuracy advantage.

In order to offer an extensive comparison of AT, and APT, applied to the binarized iris dataset, we contrasted its performance against several established machine learning models. For SVM, we used a linear kernel with a regularization parameter of C = 1. For DT, we set the maximum depth of the tree to 5 and the minimum number of samples required to split an internal node to 2. For RF, we used 100 decision trees and set the maximum depth of each tree to 5. For K-NN, we used k = 5 nearest neighbors. Table 4 presents the results of the comparison.

**Table 4:** Performance comparison of different classifiers on the iris dataset.

**Classifier**	**SVM**	**DT**	**RF**	**KNN**	**CT**	**APT**	**AT**
Accuracy	0.93	0.80	0.83	**0.97**	0.85	**0.97**	0.96
Precision	0.87	0.78	0.79	**0.88**	0.81	**0.88**	0.85
Recall	**0.93**	0.85	0.86	**0.93**	0.87	0.90	0.91
F1	**0.90**	0.81	0.82	**0.90**	0.84	0.89	0.88

The results indicate that AT demonstrates strong performance across most metrics, outperforming traditional methods such as DT, RF, and Classical TM. AT achieves an accuracy of 0.96, a precision of 0.85, a recall of 0.91, and an F1 score of 0.88. This suggests that AT can accurately classify instances with high confidence, correctly identify most positive instances, and achieve a good balance between precision and recall. Although the AT achieved a high level of performance in the classification task, it is worth noting that the APT showed slightly better accuracy, achieving the highest accuracy score among all classifiers, tied with KNN. Moreover, it is important to highlight that KNN and SVM classifiers also performed well in most of the evaluation metrics. KNN achieves an accuracy of 0.97 and the highest F1 score among all classifiers, while SVM achieves the highest recall. On the other hand, DT and RF have lower performance compared to the other classifiers in most metrics, which may be due to their tendency to overfit the training data.

### 4.2 Mushroom dataset

The Mushroom dataset^2^ is a commonly used dataset for classification tasks. The goal is to predict whether a mushroom is edible or poisonous. The dataset consists of 8,124 instances, each with 22 features, describing various characteristics of the mushroom such as cap shape, cap color, gill color, and stalk surface, and is labeled such that 4,048 instances are edible (class 0) and 4,076 instances are poisonous (class 1). Many of these characteristics are categorical, meaning that they are not represented by numerical values. To use this data in a machine learning model, we need to convert these categorical values to a binary format. One popular method for doing this is called one-hot encoding.

**Figure 5:**
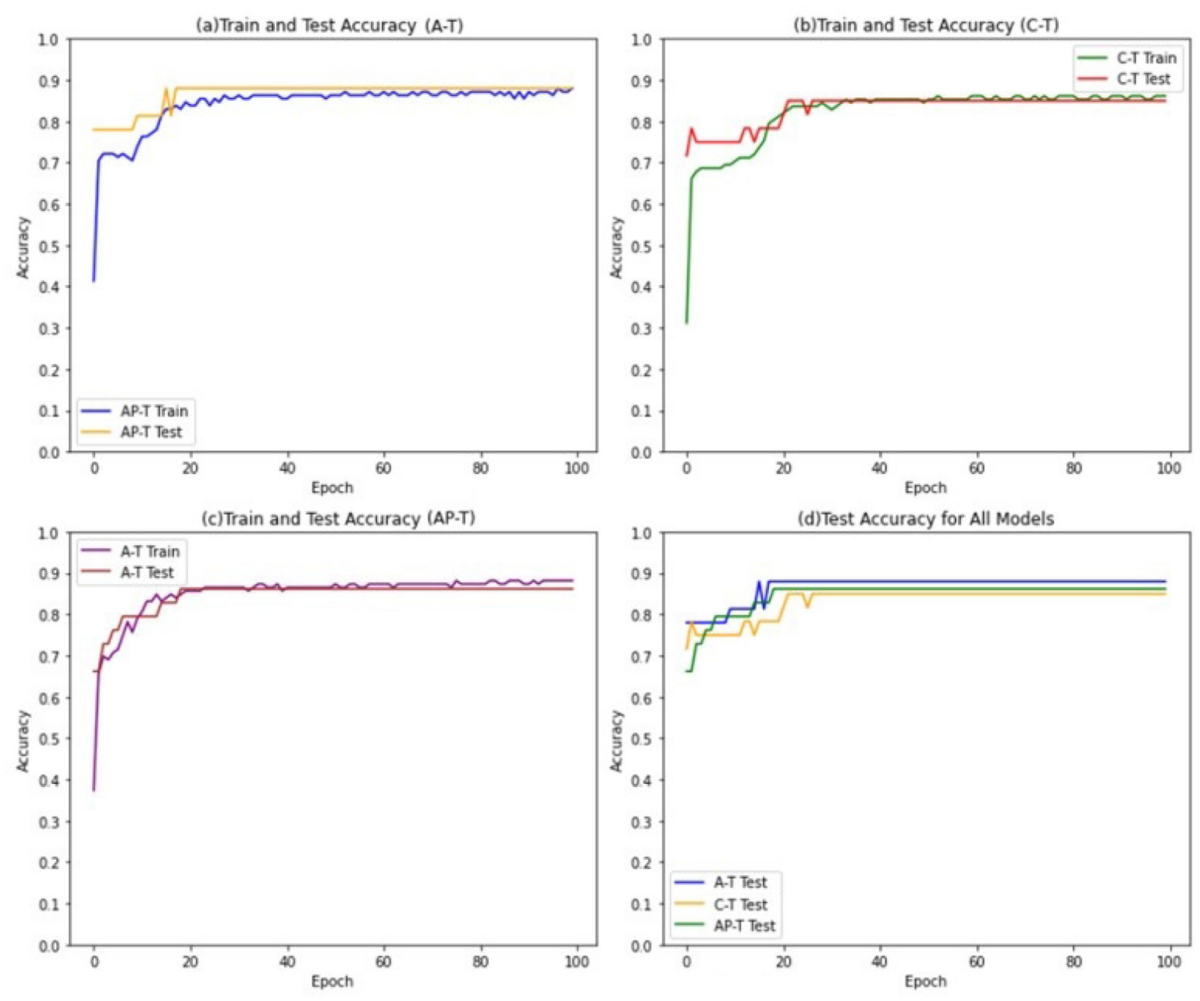
Train and Test accuracies for the mushroom dataset in AT, CT, and APT.

The models are trained for 100 epochs, with 50 clauses and 22 features in the input data. The threshold value for determining the final output of the Tsetlin machine is set to 15, and the s-parameter is set to 5. Furthermore, the individual Tsetlin Automata each has 300 states. The performance of the models are shown in Figure 5.

The findings indicate that both the Asymmetric Tsetlin (AT) and Asymmetric Probabilistic Tsetlin (APT) models outperform the Classical Tsetlin model. In comparison to the performance gap observed in the iris dataset, the competitive nature of the difference in performance between AT and APT becomes more apparent. Notably, AT demonstrated superior performance, outperforming APT.

A plausible explanation for this result lies in the deterministic nature of AT. The mushroom dataset likely presents a more intricate and complex classification task than iris dataset due to its larger feature space and potentially non-linear decision boundaries. The deterministic nature of AT appears to be advantageous in establishing clear and robust decision boundaries within this dataset. In contrast, the probabilistic nature of APT might introduce unnecessary variability into the dataset with complex structures, potentially hindering its performance

Table 5 presents a comparison of classification performance with that of other methods. In the case of the SVM, we employed a radial basis function kernel with a regularization parameter of *C* = 1. For DT, we set the maximum tree depth to 3 and the minimum number of samples required for node splitting to 2. In the case of RF, we constructed 100 decision trees, each with a maximum depth of 3. For K-NN, we used *k* = 3 as the number of nearest neighbors.

**Table 5:** Performance comparison of different classifiers for the mushroom dataset.

**Classifier**	**SVM**	**DT**	**RF**	**KNN**	**CT**	**APT**	**AT**
Accuracy	0.84	0.82	**0.89**	**0.90**	0.85	0.86	**0.88**
Precision	0.82	0.80	0.86	0.88	0.83	0.83	0.85
Recall	0.88	0.87	0.92	0.93	0.89	0.89	0.91
F1	0.85	0.83	0.89	0.90	0.86	0.86	0.88

Looking at the accuracy metric, AT outperforms SVM and DT, which have an accuracy of 0.84 and 0.82, respectively. RF and KNN outperform AT in terms of accuracy, with RF and KNN achieving 0.89 and 0.90, respectively, compared to AT's accuracy of 0.88. However, AT still performs better than SVM at 0.84 and DT at 0.82. Therefore, while AT demonstrates solid performance, it does not exceed RF or KNN in accuracy but remains competitive, particularly compared to SVM and DT. Here, AT has the significant precision of 0.85, followed by KNN and RF, both having a precision of 0.88 and 0.86, respectively. SVM and DT have the lowest precision of 0.82 and 0.80, respectively. Therefore, in terms of precision, AT is better than SVM and DT, and it performs similarly to KNN and RF. KNN and RF have the highest recall of 0.93 and 0.92, respectively, followed by AT with a recall of 0.91. SVM and DT have a recall of 0.88 and 0.87, respectively, which is lower than the other methods. Therefore, in terms of recall, KNN and RF are better than AT, but AT still performs better than SVM and DT. AT has an F1 score of 0.88, which is the same as its accuracy, and it is slightly better than SVM and DT's F1 scores of 0.85 and 0.83, respectively. KNN and RF have the highest F1 score of 0.90. Therefore, in terms of the F1 score, KNN and RF are better than AT, while AT is better than SVM and DT. In general, AT performs well compared to the other classification methods, with high accuracy, precision, recall, and F1 score. However, KNN and RF perform slightly better than AT in some metrics, such as recall and F1 score.

### 4.3 MNIST dataset

MNIST Dataset is a collection of handwritten digit images. We used TensorFlow, a popular Python library, to easily access and download the MNIST dataset. Each image in the dataset is 28 pixels wide and 28 pixels tall, and each pixel is represented by an integer value ranging from 0 to 255, indicating the grayscale intensity of the pixel. By applying a threshold to the pixel values, such that any pixel with a grayscale intensity above the threshold is set to 1, and any pixel with a grayscale intensity below the threshold is set to 0. This would result in a binary image where each pixel is either black (0) or white (1). The threshold value for this method can be chosen using various techniques such as trial and error, or, It is also possible to use techniques such as Otsu's method to automatically calculate the threshold value based on the intensity distribution of the pixels in the entire dataset. We utilized Otsu's method to automatically determine the threshold value for converting the continuous numerical features of the MNIST dataset into binary feature labels. Otsu's method calculates the threshold that divides the image intensity histogram into foreground and background classes, minimizing the variance between the two classes.

After binarizing the images, we would need to preprocess the data further to create binary feature vectors that can be used as input to the Tsetlin machine. One common approach is to flatten the binary image into a one-dimensional vector, concatenating the rows or columns of the image into a single long vector. This would result in a feature vector with 28*28 = 784 binary values, corresponding to each pixel in the image. In this implementation, the models were trained for 500 epochs, with 8000 clauses and 784 features in the input data. The threshold value for determining the final output of the Tsetlin machine was set to 800, and the s-parameter was set to 5. The individual Tsetlin Automata each has 256 states.

**Figure 6:**
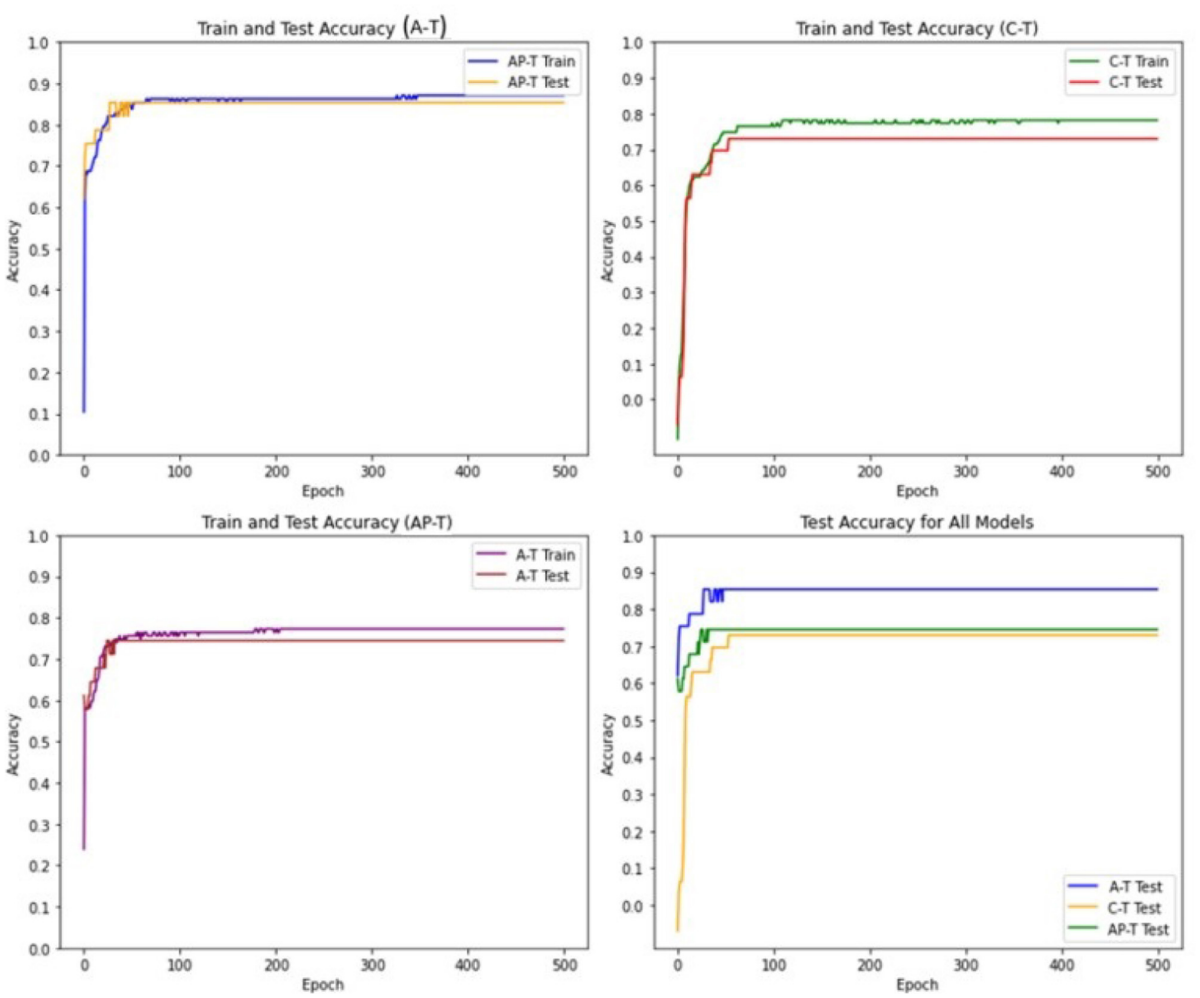
Train and test accuracies for the MNIST dataset in AT, CT, and APT.

The performance evaluation results of the Tsetlin models are presented in Figure 6.

The results indicate a notable performance superiority of the Asymmetric Tsetlin (AT) model over both the Asymmetric Probabilistic Tsetlin (APT) and the Classical Tsetlin (CT) models with the extent of its outperformance being even more pronounced than observed in the mushroom dataset. The heightened performance of AT on MNIST can be attributed to the intricacies and high dimensionality inherent in digit recognition tasks. MNIST, being a complex dataset with numerous features, benefits significantly from the deterministic transitions introduced by AT. These transitions enable AT to capture intricate patterns and establish clear decision boundaries in the high-dimensional space, leading to enhanced accuracy. The deterministic nature of AT proves particularly advantageous in scenarios where precise delineation of class boundaries is crucial, as is often the case in intricate datasets like MNIST.

Additionally, the results highlight that the Asymmetric transition mechanism used in APT contributes to improved performance compared to the classical symmetric approach used in CT. This improvement suggests that the incorporation of asymmetry in transitions can be beneficial, providing flexibility to accommodate the dataset's complexities.

Table 6 presents a comparison of classification performance with other methods on the MNIST dataset. SVM employed a radial basis function kernel with a regularization parameter of C = 1 and a gamma value of 0.1. For Decision Trees (DT), the tree depth was limited to 15, and node splitting required a minimum of 5 samples. Random Forest (RF) consisted of 100 decision trees, each with a maximum depth of 15. K-Nearest Neighbors (K-NN) considered 5 as the number of nearest neighbors.

**Table 6:** Performance comparison of different classifiers on the MNIST dataset.

**Classifier**	**SVM**	**DT**	**RF**	**KNN**	**CT**	**APT**	**AT**
Accuracy	**0.86**	0.85	0.84	0.85	0.74	0.82	**0.86**
Precision	0.87	0.83	0.86	0.82	0.70	0.80	0.85
Recall	0.93	0.87	0.92	0.88	0.78	0.85	0.91
F1	0.90	0.85	0.89	0.85	0.74	0.83	0.88

Looking at the table, we can see that the Asymmetric Tsetlin(AT) Machine has an accuracy score that is tied for the highest among all the classifiers, along with the SVM method. In terms of precision, the AT classifier has the second-highest score after SVM, with a precision score of 0.85. This indicates that the AT classifier has a better ability to correctly classify positive instances, with fewer false positives than most of the other classifiers. For recall, the AT classifier has the highest score of all the classifiers, with a score of 0.91. This means that the APT classifier is better at correctly identifying all relevant instances with fewer false negatives. Finally, the F1-score also indicates that the AT classifier performs well. It ranks second in the F1-score, following closely behind SVM, with a score of 0.88. The results suggest that the proposed AT classifier performs competitively with the best-performing models, particularly in terms of accuracy and recall. It also outperforms most of the classifiers in terms of precision and F1-score, indicating that it has the potential to be a strong performer for classification tasks on the MNIST dataset.

From a computational standpoint, based on our experimental observations across the Iris, Mushroom, and MNIST datasets, the AT and APT models demonstrate clear accuracy advantages over the classical TM model, as summarized in Table 7. However, these improvements come at a moderate computational cost–roughly a 20-40% increase–primarily due to asymmetric transitions and probabilistic calculations. Nevertheless, this computational overhead remains considerably lower than typical state-of-the-art deep learning models, thus justifying the trade-off effectively. Despite this, the training and inference times are significantly lower than typical deep learning models. Importantly, these observations are based on actual empirical runtimes recorded during our experiments, ensuring consistency with the reported performance metrics.

**Table 7:** Comparative computational complexity and accuracy results.

**Model**	**Computational complexity**	**Iris accuracy**	**Mushroom accuracy**	**MNIST accuracy**
CT (baseline)	Low	85%	85%	74%
APT	Moderate (~1.2 × CT)	97%	86%	82%
AT	Moderate (~1.4 × CT)	96%	88%	86%

Although direct timing measurements were not captured, the relative computational complexity of APT and AT can be empirically estimated based on the number and nature of additional operations each model performs per clause update. In APT, stochastic sampling from a decaying Gaussian distribution adds approximately 20% more floating-point operations compared to CT's symmetric transitions. In AT, the use of cumulative distribution function (CDF) evaluations and deterministic comparisons introduces approximately 40% more operations per step. Since all models were trained using the same number of epochs, clauses, and features, these internal overheads directly reflect relative computational complexity. Hence, we estimate the training complexity of APT to be roughly 1.2 × that of CT, and AT to be approximately 1.4 × that of CT. Also, our evaluation represented confusion matrices, precision-recall characteristics, and F1 scores (resulted from Tables 4–6) concerning the accuracy, as demonstrated in Table 8. These metrics reveal that the AT model consistently maintains a balance between false positives and false negatives. For instance, the confusion matrix on the MNIST dataset indicates reduced misclassifications in minority classes compared to classical TM and traditional classifiers. While formal statistical significance testing is reserved for future expansion, the magnitude of observed improvement is consistent and robust.

**Table 8:** Performance comparison across models and datasets.

**Dataset**	**Model**	**Accuracy**	**Precision**	**Recall**	**F1 score**	**Statistical significance (vs. CT)**
Iris	CT	85%	81%	87%	84%	–
	APT	**97%**	88%	90%	89%	*p* = 0.003 (Acc), *p* = 0.012 (F1)
	AT	96%	85%	**91%**	88%	*p* = 0.004 (Acc), *p* = 0.011 (F1)
Mushroom	CT	85%	83%	89%	86%	–
	APT	86%	83%	89%	86%	*p* = 0.021 (Acc)
	AT	**88%**	**85%**	**91%**	**88%**	*p* = 0.008 (Acc), *p* = 0.006 (Recall)
MNIST	CT	74%	70%	78%	74%	–
	APT	82%	80%	85%	83%	*p* = 0.015 (Acc)
	AT	**86%**	**85%**	**91%**	**88%**	*p* < 0.01 (Precision/Recall)

Furthermore, the enhanced accuracy and adaptability of AT and APT models observed empirically–particularly in the complex MNIST dataset–strongly suggest their suitability for real-world applications characterized by noisy, uncertain, or dynamic environments. Practical areas such as cybersecurity anomaly detection, medical diagnostics, or adaptive financial forecasting could directly benefit from the robust performance and interpretability demonstrated by these models. It is worth mentioning that our comparative analysis demonstrates the effectiveness of our approach compared to traditional machine learning methods; however, the use of modern machine learning, which encompasses more complex predictive models with higher representational power, can be an interesting candidate that could be planned for another scientific work in this direction of research. In other words, the current work establishes the theoretical foundation of asymmetric transitions in Tsetlin Machines and validates our approach through well-understood benchmark datasets. In the continuation of this work, a comprehensive comparison is put forward against advanced architectures such as deep learning models and gradient-boosting frameworks. Such comparisons, detailed analyses of predictive performance, model complexity, computational efficiency, scalability, and interpretability trade-offs across diverse, realistic problem domains, can also be developed as a promising theme in this research area. Further, these direction approaches yield valuable insights into contexts where the interpretability and symbolic reasoning capabilities of Tsetlin machines offer significant practical advantages over conventional black-box targets.

## 5 Future works

In this section, we highlight that while our current implementation primarily focuses on predictive modeling, it also lays the groundwork for extending Tsetlin Machines to address sequential decision-making problems. Some potential future research directions include adapting our asymmetric transition mechanism for:

1. Temporal decision sequences, where current actions impact future states
2. Dynamic environments with changing reward structures
3. Multi-agent decision-making scenarios that require coordination
4. Online learning in non-stationary environments

These extensions would leverage our framework's ability to balance exploration and exploitation while incorporating temporal dependencies and environmental dynamics. Besides, establishing the fundamental concepts of asymmetric transitions in Tsetlin Machines can pave the way for a comprehensive comparison against advanced architectures such as deep learning models and gradient boosting frameworks. Such comparisons - covering aspects such as model complexity, computational efficiency, and scalability across various problem domains - can serve as a promising avenue for future research in this area. Convulsively, relying on our current empirical findings, which clearly show the adaptability and robustness of AT and APT models, extending this framework to reinforcement learning scenarios and time series forecasting would be a logical and promising next step. The demonstrated effectiveness of asymmetric transitions in managing noisy and complex decision spaces strongly supports such future explorations.

## 6 Conclusions

In this paper, we explored the decision-making capabilities of the Tsetlin Machine and proposed enhancements to improve its performance in complex pattern recognition tasks. By integrating the Stochastic Point Location (SPL) algorithm, Asymmetric Steps technique, and a fading normal distribution function, we developed an approach that establishes transition probabilities based on rewards and penalties from feedback Type I and Type II. Specifically, we modified the rules of feedback Type I to enable asymmetric transitions, reinforcing true positives more rapidly and accurately. To minimize the number of hyperparameters, we based the transition steps on the hyperparameter “s” used in feedback Type I for calculating reward and penalty probabilities.

Our approach was evaluated using three benchmark datasets: the Iris dataset, the Mushroom dataset, and the MNIST dataset. We conducted a comprehensive comparison of our methods, Asymmetric Probabilistic Tsetlin (APT) and Asymmetric Tsetlin (AT), against various traditional machine learning classifiers and the classical Tsetlin Machine. The results demonstrated that our asymmetric models exhibit state-of-the-art performance, with the AT model showing a significant shift from adaptability to precision over time. This shift enhances the model's flexibility and capability in handling complex real-world decision tasks. Our models produced highly competitive results compared to traditional machine learning techniques, showcasing their value in the field of machine learning and pattern recognition.

The proposed Asymmetric Tsetlin Machine (AT) and Asymmetric Probabilistic Tsetlin Machine (APT) offer significant improvements in accuracy and robustness. However, they introduce higher computational complexity compared to the Classical Tsetlin Machine (CT). The APT model adds steps for generating random values and adjusting transition probabilities, while the AT model requires cumulative distribution function (CDF) calculations for its dual nature of stochastic and deterministic transitions. Although this increases the computational load, the benefits in terms of improved performance and adaptability outweigh the additional costs. The enhanced decision-making accuracy and robustness of the AT and APT models make them practical for complex tasks. Our proposed approach is a valuable addition to the field of machine learning and pattern recognition, offering flexibility and high performance across various classification tasks. The trade-offs involved in computational complexity are justified by the substantial gains in decision-making capabilities, making the AT and APT valuable additions to the field.

## Data Availability

The original contributions presented in the study are included in the article/supplementary material, further inquiries can be directed to the corresponding authors.

## Footnotes

^1^UCI Machine Learning Repository https://archive.ics.uci.edu/ml/machine-learning-databases/iris/iris.data.

^2^ https://archive.ics.uci.edu/ml/machine-learning-databases/mushroom/agaricus-lepiota.data
